# Physician Estimates and Patient-Reported Health Status in Atrial Fibrillation

**DOI:** 10.1001/jamanetworkopen.2023.56693

**Published:** 2024-02-23

**Authors:** Nobuhiro Ikemura, Shun Kohsaka, Takehiro Kimura, Philip G. Jones, Yoshinori Katsumata, Kojiro Tanimoto, Ikuko Ueda, Seiji Takatsuki, Masaki Ieda, Paul S. Chan, John A. Spertus

**Affiliations:** 1 Department of Cardiology, Keio University School of Medicine, Tokyo, Japan; 2 University of Missouri–Kansas City’s Healthcare Institute for Innovations in Quality and Saint Luke’s Mid America Heart Institute, Kansas City; 3 Department of Cardiology, National Hospital Organization, Tokyo Medical Center, Tokyo, Japan

## Abstract

**Importance:**

A primary objective in managing atrial fibrillation (AF) is to optimize patients’ health status, which can be done only if physicians accurately quantify the outcomes associated with AF in patients’ lives.

**Objective:**

To explore physicians’ estimation of the health status of patients with AF and its association with subsequent care and outcomes.

**Design, Setting, and Participants:**

A multicenter, prospective cohort study was conducted in 2 outpatient practices in Tokyo, Japan. Participants included patients with newly diagnosed AF or those referred for initial treatment of AF at outpatient practices and treating physicians from November 8, 2018, to April 1, 2020. Data analysis was performed from December 22, 2022, to July 7, 2023.

**Exposures:**

Participating patients completed the Atrial Fibrillation Effect on Quality-of-Life (AFEQT) questionnaire, a 20-item tool covering 4 domains with a 7-point Likert scale; 3 domains (symptoms, daily activities, and treatment concerns) were used in this study. Blinded to patients’ responses, treating physicians answered a 3-item questionnaire quantifying each patient’s AFEQT domain with a single item. Patients’ mean Likert scale responses within each AFEQT domain were subtracted from the physicians’ assessments so that higher scores (≥0.5 points) indicate physician underestimation, while lower scores (≤0.5 points) indicate physician overestimation of the health status of patients with AF.

**Main Outcomes and Measures:**

The independent association of physician-patient concordance with treatment escalation (alteration or initiation of antiarrhythmic drugs, cardioversion, or catheter ablation) and 1-year adjusted changes in AFEQT scores.

**Results:**

Among 330 patients (238 [72.1%] men; mean [SD] age, 67.9 [11.9] years; 163 [49.4%] with paroxysmal AF), physicians correctly estimated health status in 112 patients (33.9%), underestimated it in 42 patients (12.7%), and overestimated it in 176 patients (53.3%). Treatment escalation occurred in 63.6% of patients whose health status was correctly estimated, 47.6% of those whose health status was underestimated, and 66.3% of patients whose health status was overestimated. After multivariable adjustment, underestimation of health status was independently associated with less treatment escalation (adjusted odds ratio, 0.43; 95% CI, 0.20-0.90) and less frequent AFEQT overall summary score improvement at 1 year (underestimated, 2.5 [95% CI, −1.6 to 6.7] vs correctly and overestimated health status, 8.4 [95% CI, 7.0-9.9] points; *P* = .01).

**Conclusions and Relevance:**

In this cohort study, physician underestimation of the health status of patients with AF was common and associated with less aggressive treatment and less health status improvement at 1 year.

## Key Points

**Question:**

Do physicians correctly recognize the health status of patients with atrial fibrillation (AF)?

**Findings:**

This cohort study in 330 patients with AF found that physician underestimation or overestimation of the health status of patients with AF was common in routine clinical practice. Physician underestimation was associated with less aggressive treatment and smaller 1-year improvements in patients’ health status.

**Meaning:**

The findings of this study suggest that standardized assessments of patients’ health status with validated questionnaires may help reduce inaccurate estimations of specific health status of patients with AF, resulting in more appropriate treatment and better outcomes.

## Teaser

This cohort study examines physician estimation of the health status of patients with atrial fibrillation and changes in treatment following the estimation.

## Introduction

Atrial fibrillation (AF) is the most common sustained arrhythmia and is increasing in prevalence throughout the world.^1^ As the health status of patients with AF is frequently impaired, optimizing their symptoms, function, and quality of life is a primary treatment goal for managing AF in the outpatient setting.^2,3^ Various treatments, including rate-controlling medications and restoration of sinus rhythm (eg, cardioversion or catheter ablation), can improve patients’ health status.^2,4^ However, the foundation of effectively treating patients’ health status is to accurately quantify it.

Recognizing the severity of patients’ health status can be challenging due to variability in history taking and time constraints faced by both patients and physicians. In prior work, 27% of patients with impaired health status due to AF were labeled as asymptomatic by physicians in their medical records, resulting in a lower likelihood of patients undergoing catheter ablation therapy.^5,6^ This study also revealed a high rate of overestimation of symptoms, with 45% of patients reporting no health status impairments, but being labeled as having symptomatic AF in their medical records. While the high prevalence of underestimation and overestimation of AF-related health status is concerning, a major limitation of this previous report was the use of dichotomized physician assessments of symptoms based on medical records, thereby oversimplifying physicians’ estimates of patients’ symptoms and potentially introducing misclassification.

To better describe potential discrepancies in health status assessment between patients and physicians, we prospectively obtained health status assessments from both patients and their physicians to explore the extent of underestimation or overestimation of the health status of patients with AF by physicians. We then examined whether physician underestimation or overestimation of the patients’ health status was associated with differences in treatment escalation and changes in patients’ reported health status at 1 year. Identifying lower rates of treatment escalation among patients whose health status was underestimated may indicate missed opportunities to optimize the use of potentially beneficial therapies and could support the collection of information on patients’ health status in routine clinical practice.

## Methods

### Data Sources

We enrolled patients with AF who were newly diagnosed or referred to a cardiology outpatient clinic at 2 tertiary care hospitals in Tokyo, Japan (Keio University Hospital and Tokyo Medical Center), between November 8, 2018, and April 1, 2020. We screened patients from lists provided monthly by each hospital, and a query was sent for study participation if physicians set an appointment with patients under the new diagnostic coding of AF. The diagnosis of AF was confirmed by the treating physician based on electrocardiographic documentation (eg, electrocardiogram, Holter monitoring, and implanted device); patients with atrial flutter alone were not eligible. The rationale for limiting participants to newly diagnosed AF or those referred for initial AF evaluation was to obtain patient data before treatment initiation. This is a substudy from the previously described Keio Interhospital Studies–Atrial Fibrillation registry.^7^ In brief, trained study coordinators abstracted approximately 150 variables regarding patients’ sociodemographic characteristics, comorbidities, vital signs, and anthropomorphic data; current and prior medication use; electrocardiographic and echocardiographic data; and routine blood test results.^7^ On-site auditing was performed to ensure consecutive case enrollment. Annual follow-up assessments for up to 2 years were performed by mail, telephone interviews, and medical records review.^7^ Data quality assurance was achieved through systematic validation that highlighted outliers and data completeness.^7^ The protocol was approved by the ethical review board of each institution, and all participants provided written informed consent; no financial compensation was provided. This study followed the Strengthening the Reporting of Observational Studies in Epidemiology (STROBE) reporting guideline as well as the Standards for Quality Improvement Reporting Excellence (SQUIRE) reporting guideline for quality improvement studies.^8^

### Health Status Assessments

Patient-reported health status, using the validated Atrial Fibrillation Effect on Quality-of-Life (AFEQT) questionnaire, was collected at enrollment and at 1 year.^9^ The AFEQT is a 20-item questionnaire that quantifies AF-related health status in 4 domains that are established by factor analysis: symptoms (Q1-4), daily activities (Q5-12), treatment concerns (Q13-18), and treatment satisfaction (Q19-20), using a 7-point Likert response scale, with higher scores denoting worse health status. A culturally and linguistically translated version of the AFEQT questionnaire for Japan was used. For this study, we focused on the first 3 domains of patients’ health status: symptoms, daily activities, and treatment concerns, which are combined to create an AFEQT Overall Summary (AFEQT-OS) score.

Blinded to patients’ AFEQT responses and immediately following the visit, treating physicians at the 2 participating sites were asked to assess whether patients had experienced any difficulties due to AF in the previous month. Using a 3-item questionnaire to reduce the burden (eAppendix in Supplement 1), participating physicians provided assessments based on their clinical judgment regarding how AF affected patients’ symptoms (QI), daily activities (QII), and treatment concerns (QIII), using a similar 7-point Likert scale rating, with higher scores denoting worse health status as used in the AFEQT. To account for the difference in the questionnaire between patients and physicians, we ensured that patients’ responses were not correlated with physicians’ estimations by identifying that the overall correlations were modest. This allowed a direct comparison between patients’ and physicians’ assessments of each AFEQT health status domain by averaging the patients’ responses to items in each domain and comparing them with the physicians’ assessments.

### Assessing Physician and Patient Concordance and Discordance in Patients’ Health Status

The patients’ health status assessments, as measured by the AFEQT questionnaire, were considered the reference standard. To assess physicians’ overestimation and underestimation of the health status of patients with AF, we first computed patients’ mean Likert response to each of the 3 AFEQT domains. We then computed the mean of these 3 domains to generate a patient’s overall mean Likert score rating (range: 1-7, with higher scores indicating worse health status) and compared them with the mean of physicians’ evaluations on the 3 domains (QI-III).

To objectively define clinically meaningful differences in the mean Likert score ratings between physicians and patients, we assessed the patients’ overall mean Likert score and their overall AFEQT score (AFEQT-OS). As the AFEQT-OS score ranges from 0 (indicating the worst possible health state) to 100 (the best possible health state), each 1-point difference in a patient’s overall mean Likert score corresponds to a 16.6-point difference in AFEQT-OS scores (eFigure 1 in Supplement 1). In prior work, a 7.2-point improvement or worsening of the AFEQT-OS score was determined to be a clinically important difference in patients’ health status, defined as a shift of one class in the European Heart Rhythm Association classification, for example, from class II (eg, mild to moderate symptoms, normal daily activities not affected), to class III (eg, severe symptoms, normal daily activities affected).^10^ The shift corresponded to a 0.43-point change in patients’ overall mean Likert score rating. Therefore, in this study, we divided patients into 3 groups according to the differences in overall mean Likert scores between physicians and patients using a 0.5-point threshold, representing an 8.4-point difference in AFEQT-OS scores: (1) a correctly estimated group, where the difference in mean was less than 0.5; (2) an underestimated group, where the physician’s score was greater than or equal to 0.5 points lower than the patient’s score (suggesting physician underestimation); and (3) an overestimated group, where the physician’s score was greater than or equal to 0.5 points higher than the patient’s score (suggesting physician overestimation).

### Outcomes Definition

An additional goal of this study was to examine whether physician underestimation or overestimation of the health status of patients with AF was associated with differences in treatment escalation by the physician within 1 year of presentation. Any of the following clinical decisions were considered treatment escalation: (1) alteration or initiation of antiarrhythmic drug therapy, (2) referral for electrical or pharmacologic cardioversion, and (3) catheter ablation for AF. Antiarrhythmic drugs included procainamide, quinidine, cibenzoline, disopyramide, aprindine, pilsicainide, flecainide, propafenone, amiodarone, and bepridil. We did not consider rate control medications (eg, β-blockers, calcium channel blockers, and digitalis) as treatment escalation, since these medications are also used to manage other cardiac conditions (eg, hypertension, coronary artery disease, and heart failure) and focused on strategies for rhythm restoration. The clinical research coordinators reviewed the change or initiation of antiarrhythmic drugs and physicians’ orders for procedures at annual follow-up assessments by medical records review, mail, and telephone interviews. The number of outpatient visits and follow-up periods after initial enrollment were left to each physician’s discretion. Physicians were blinded to the patients’ AFEQT results both at enrollment and at 1-year follow-up.

### Statistical Analysis

Data analysis was performed from December 22, 2022, to July 7, 2023. We describe the distribution of both patient’s reported and physician’s estimation of health status. To account for the difference in the questionnaire between patients and physicians, we explored variability in both responses to the different questionnaires using Spearman rank correlation coefficients.

Patients’ demographic and clinical characteristics were compared between those whose physicians underestimated, correctly estimated, and overestimated the health status of patients with AF using 1-way analysis of covariance for continuous variables and χ^2^ tests for categorical variables. The AFEQT scores at baseline and 1-year follow-up were also calculated and compared between the 3 groups. To evaluate the association between physician-patient concordance with changes in patients’ reported health status, 1-year changes in AFEQT scores were compared using analysis of covariance adjusted for baseline AFEQT scores, where positive changes indicate improved health status and negative changes indicate worsening health status. Additionally, we constructed a hierarchical linear regression model to evaluate the independent associations between physician-patient health status concordance (underestimated vs correctly and overestimated) and changes in AFEQT scores, adjusting for site as a random effect to account for within-hospital practice patterns. We also adjusted for clinically relevant variables as fixed effects, including age (≥75 vs <75 years), sex, prior heart failure, type of AF (paroxysmal vs other), use of antiarrhythmic drugs at enrollment, history of catheter ablation for AF, baseline AFEQT-OS score, and CHA_2_DS_2_-VASc (cardiac failure or dysfunction, hypertension, age 75 years [doubled], diabetes, stroke [doubled]–vascular disease, age 65-74 years, and sex category [female]) score. Furthermore, we constructed a hierarchical logistic regression model to evaluate the independent associations between physician-patient health status concordance and treatment escalation, adjusting for all variables included in the hierarchical linear regression model as fixed effects and site as a random effect. There were no missing data in the studied variables.

SPSS, version 29.0 (IBM Corp) and R statistical software, version 4.1.2 (R Foundation for Statistical Computing) were used for analyses. All reported *P* values are 2-sided, with differences at *P* < .05 being considered statistically significant.

## Results

### Study Sample

**Figure 1.**
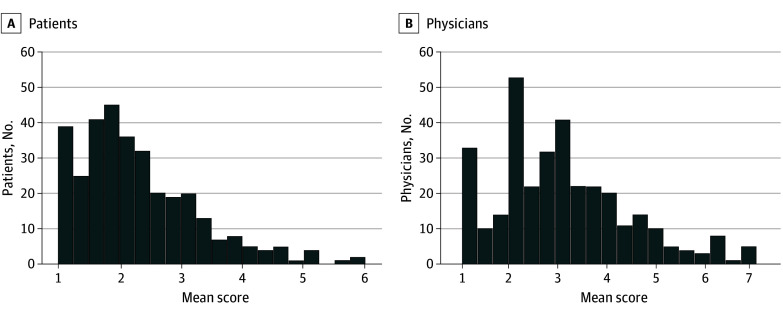
Distribution of Patients' Mean Raw Responses and Physicians' Mean Estimations A higher score indicates worse health status.

Overall, 330 consecutive outpatients with AF were enrolled from 7 physicians. There were no exclusions due to missing health status evaluations from either patients or physicians. The mean (SD) age of all patients was 67.9 (11.9) years; 238 (72.1%) were men, 92 (27.9%) were women, 163 (49.4%) had paroxysmal AF, the mean CHA_2_DS_2_-VASc score was 2.3 (1.5), and the mean HAS-BLED (hypertension, abnormal kidney/liver function, stroke, bleeding history or predisposition, labile international normalized ratio, elderly, drugs/alcohol concomitantly) score was 1.1 (0.8). Kidney function was measured using the Japanese equation for estimated glomerular filtration rate.^11^ The overall distributions of the mean Likert scores from patients and physicians are shown in Figure 1, and the 3 health status domains are shown in eFigure 2 in Supplement 1, suggesting a shift toward higher ratings among physicians.

### Correlation Between Patients’ Reported Health Status and Physicians’ Estimation

Correlations between patients’ reported health status on the Likert scale from 1 to 7 and physicians’ estimation are summarized in Table 1. The mean (SD) Likert score rating for each of the 3 main AFEQT domains (symptoms, daily activity, and treatment concerns) were 2.1 (1.0) for symptoms, 2.2 (1.2) for daily activity, and 2.4 (1.6) for treatment concerns for patients, and 3.3 (1.5) for symptoms, 2.6 (1.5) for daily activity, and 3.3 (1.4) for treatment concerns for physicians. The correlations between patients’ 1 to 7 Likert scale ratings for individual AFEQT items and physicians’ estimations in the same domains were modest (*r* value range, 0.33-0.49). Higher positive correlations were observed with symptoms and daily activities than with treatment concerns. The strongest correlations between physicians’ estimations of symptoms were with patients’ reports of palpitations (*r* = 0.47) and irregular heartbeat (*r* = 0.47). Similarly, physicians’ estimation of the difficulties in daily activities were most strongly associated with patients’ reports of fatigue (*r* = 0.49) and shortness of breath (*r* = 0.48) with activity and with exercising on the AFEQT. The correlations between patients’ and physicians’ estimation of AF-related treatment concerns were low across all patient-reported items.

**Table 1.** Correlations Between Patients’ Reported Health Status and Physicians’ Estimation

Questions for physician	Correlation coefficient^a^	*P* value
**How much do you think this patient has been suffering from symptoms of atrial fibrillation?**		
As a result of your atrial fibrillation, how much were you bothered by		
Q1: palpitations: heart fluttering, skipping, or racing	0.47	<.001
Q2: irregular heartbeat	0.47	<.001
Q3: a pause in heart activity	0.25	<.001
Q4: lightheadedness or dizziness	0.27	<.001
**How much do you think this patient’s daily activity is limited by atrial fibrillation?**		
Have you been limited by your atrial fibrillation in your		
Q5: ability to have recreational pastimes, sports, and hobbies	0.33	<.001
Q6: ability to have a relationship and do things with friends and family	0.36	<.001
As a result of your atrial fibrillation, how much difficulty have you had in		
Q7: doing any activity because you felt tired, fatigued, or low on energy	0.49	<.001
Q8: doing physical activity because of shortness of breath	0.48	<.001
Q9: exercising	0.47	<.001
Q10: walking briskly	0.42	<.001
Q11: walking briskly uphill or carrying groceries or other items, up a flight of stairs without stopping	0.42	<.001
Q12: doing vigorous activities, such as lifting or moving heavy furniture, running, or participating in strenuous sports like tennis or racquetball	0.40	<.001
**How anxious do you think this patient is about atrial fibrillation and its treatment?**		
As a result of your atrial fibrillation, how much did the feelings below bother you?		
Q13: feeling worried or anxious that your atrial fibrillation can start anytime	0.31	<.001
Q14: feeling worried that atrial fibrillation may worsen other medical conditions in the long run	0.34	<.001
As a result of your atrial fibrillation treatment, how much were you bothered by		
Q15: worrying about the treatment side effects from medications	0.24	<.001
Q16: worrying about complications or side effects from procedures like catheter ablation, surgery, or pacemaker therapy	0.24	<.001
Q17: worrying about side effects of blood thinners, such as nosebleeds, bleeding gums when brushing teeth, heavy bleeding from cuts, or bruising	0.13	.03
Q18: worrying or feeling anxious that your treatment interferes with your daily activities	0.19	.001

^a^ Range, 0 to 1; a higher number indicates more agreement between patients and physicians.

### Baseline Characteristics Associated With the Concordance and Discordance of Physicians’ Assessments

**Figure 2.**
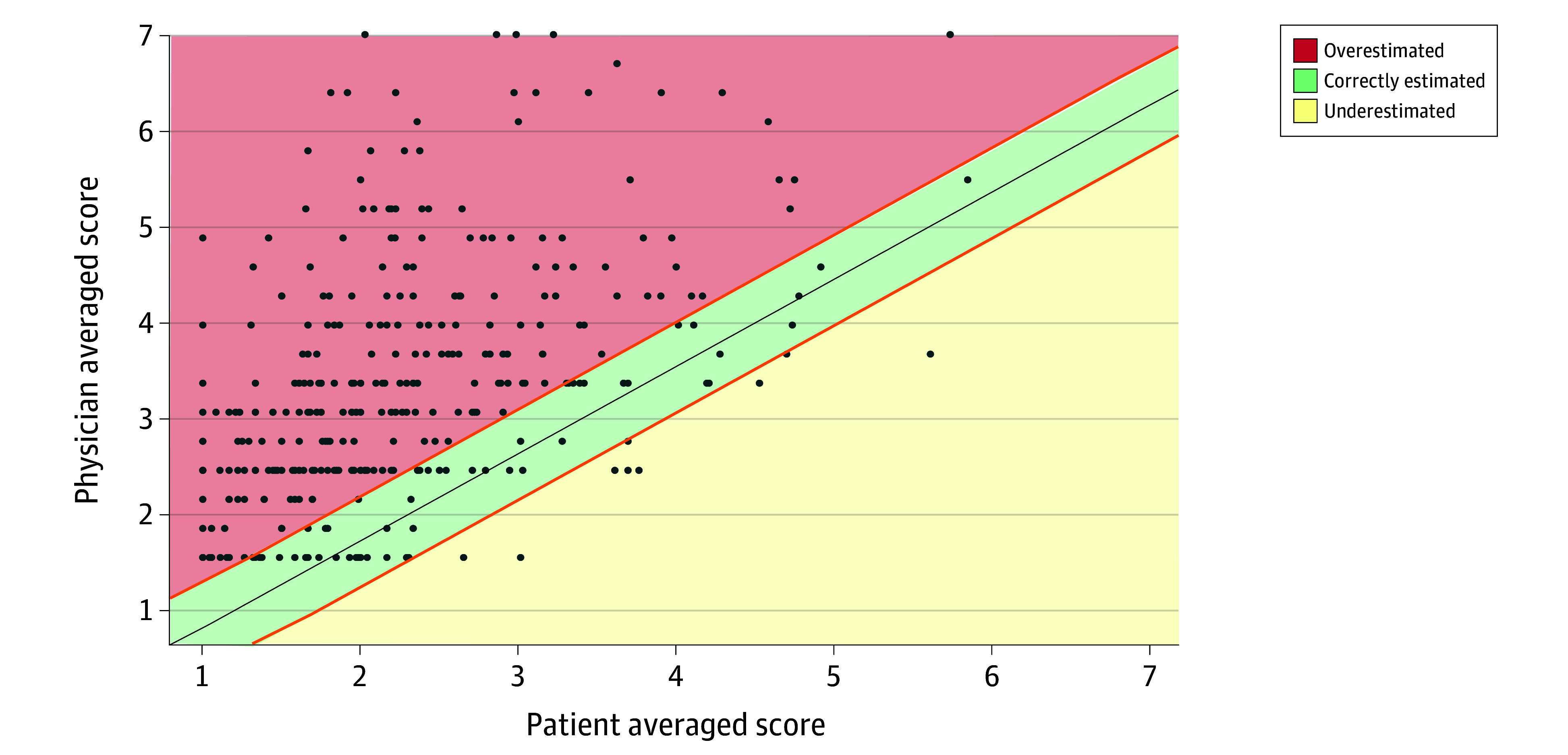
Overall Distribution for Both Patients’ and Physicians’ Overall Mean Likert Scores A higher score indicates worse health status. The center gray line indicates the ideal concordance between patients and physicians, and the outer orange lines indicate the threshold of 0.5 points for overestimation and underestimation.

There were 112 patients (33.9%) whose health status was correctly estimated by physicians, 42 patients (12.7%) whose health status was underestimated, and 176 (53.3%) whose health status was overestimated it (Figure 2). Clinical characteristics across 3 groups are summarized in Table 2. Patients whose health status was underestimated were more likely to be younger (mean [SD] age, 63.7 [10.6] vs 65.6 [12.3] years) and have a history of AF ablation (8 of 42 [19.0%] vs 9 of 112 [8.0%]) than in the correctly estimated group. Patients whose physicians overestimated their health status were more likely to be older (mean age, 70.4 [11.5] vs 65.6 [12.3] years) than those in the correctly estimated group. There were no significant differences in the type of AF across the 3 groups, but the underestimated and overestimated groups were more likely to include more females, have a history of heart failure, be treated with β-blockers or diuretics, and have had a higher B-type natriuretic peptide level than the correctly estimated group.

**Table 2.** Baseline Characteristics

Characteristic	No. (%)			*P* value
	Underestimated^a^	Correctly estimated	Overestimated^b^	
Patients	42 (12.7)	112 (33.9)	176 (53.3)	NA
Age, mean (SD), y	63.7 (10.6)	65.6 (12.3)	70.4 (11.5)	.002
Sex				
Men	32 (76.2)	90 (80.4)	116 (65.9)	.02
Women	10 (23.8)	22 (19.6)	60 (34.1)	
BMI, median (SD)	24.4 (2.6)	24.3 (3.5)	24 (4.1)	.28
Medical history				
Smoking	13 (31.0)	18 (16.1)	32 (18.2)	.10
Hypertension	24 (57.1)	62 (55.4)	110 (62.5)	.46
Diabetes	6 (14.3)	18 (16.1)	21 (11.9)	.60
Dyslipidemia	21 (50.0)	41 (36.6)	80 (45.5)	.20
Heart failure	4 (9.5)	4 (3.6)	21 (11.9)	.05
Stroke or TIA	4 (9.5)	9 (8.0)	20 (11.4)	.87
CKD^c^	24 (57.1)	42 (38.2)	82 (47.7)	.08
Peripheral artery disease	3 (7.1)	6 (5.4)	6 (3.4)	.51
Coronary artery disease	1 (2.4)	5 (4.5)	6 (3.4)	.80
CHA_2_DS_2_-VASc score, mean (SD)	2 (1.5)	2 (1.4)	2.6 (1.5)	.006
HAS-BLED score, mean (SD)	1 (1.0)	0.9 (0.8)	1.2 (0.8)	.006
Type of visit				
Referral from emergency department	1 (2.4)	4 (3.6)	9 (5.1)	.66
Diagnosed at health screening	10 (23.8)	45 (40.2)	52 (29.5)	.07
Type of AF at registration				
First detected	1 (2.4)	8 (7.1)	10 (5.7)	.63
Paroxysmal	19 (45.2)	53 (47.3)	91 (51.7)	
Persistent	20 (47.6)	40 (35.7)	60 (34.1)	
Permanent	2 (4.8)	11 (9.8)	15 (8.5)	
Prior catheter ablation	8 (19.0)	9 (8.0)	15 (8.5)	.09
Current drug therapy				
β-Blockers	21 (50.0)	47 (42.0)	104 (59.1)	.01
ACE inhibitors/ARBs	20 (47.6)	35 (31.3)	59 (33.5)	.15
Calcium channel blockers	12 (28.6)	52 (46.4)	86 (48.9)	.06
Digoxin	1 (2.4)	2 (1.8)	1 (.6)	.49
Diuretics	7 (16.7)	12 (10.7)	31 (17.6)	.27
Currently using antiarrhythmic drugs	10 (23.8)	16 (14.3)	26 (14.8)	.30
Oral anticoagulants				
None	6 (14.3)	19 (17.0)	20 (11.4)	.39
Warfarin	0 (.0)	2 (1.8)	6 (3.4)	.37
Direct oral anticoagulants	36 (85.7)	91 (81.2)	150 (85.2)	.53
Antiplatelet therapy	8 (19.0)	6 (5.4)	12 (6.8)	.01
BNP, mean (SD), pg/mL	116.5 (134.1)	105.3 (99.1)	180.4 (256.7)	.01
AFEQT questionnaire score, mean (SD)				
Overall summary	65.7 (18.9)	81.9 (13.4)	81.3 (13.5)	<.001
Symptoms	73.1 (21.5)	86.0 (13.0)	83.0 (14.5)	<.001
Daily activities	62.5 (23.4)	82.5 (16.9)	81.5 (18.7)	<.001
Treatment concern	65.1 (18.9)	78.3 (17.5)	80.6 (14.0)	<.001

Abbreviations: ACE, angiotensin-converting enzyme; AF, atrial fibrillation; AFEQT, Atrial Fibrillation Effect on Quality-of-Life; ARBs, angiotensin receptor blockers; BMI, body mass index (calculated as weight in kilograms divided by height in meters squared); BNP, brain natriuretic peptide; CHA_2_DS_2_-VASc, cardiac failure or dysfunction, hypertension, age 75 years (doubled), diabetes, stroke (doubled)–vascular disease, age 65-74 years, and sex category (female); CKD, chronic kidney disease; eGFR estimated glomerular filtration rate; HAS-BLED, hypertension, abnormal kidney/liver function, stroke, bleeding history or predisposition, labile international normalized ratio, elderly, drugs/alcohol concomitantly; NA, not applicable; TIA, transient ischemic attack.

SI conversion factor: To convert BNP to nanograms per liter, multiply by 1.

^a^ Denotes physician underestimation of the health status of patients with AF.

^b^ Denotes physician overestimation of the health status of patients with AF.

^c^ Chronic kidney disease considered eGFR less than 60 mL/min/1.73 m^2^, calculated based on the formula eGFR = 194 × serum creatinine (mg/dL)^−1.094^ × age^−0.287^ × 0.739 (if female).^11^ To convert serum creatinine to micromoles per liter, multiply by 88.4.

### Health Status Outcomes at Baseline and 1-Year Follow-Up

The mean baseline AFEQT-OS score across these 3 groups was 65.7 (18.9) in the underestimated group, 81.9 (13.4) in the correctly estimated group, and 81.3 (13.4) in the overestimated group. The underestimated group had lower symptom and daily activities scores (Table 2).

At 1-year follow-up, 290 patients (88.1%) completed the AFEQT questionnaire. The group in which physicians underestimated the health status exhibited significantly lower scores in the AFEQT-OS than the other 2 groups (underestimated, 81.2 [17.8]; correctly estimated, 90.1 [11.4]; and overestimated, 85.8 [14.1]; *P* < .001) (Table 3), driven primarily by lower scores in the symptom and daily activities domains. After adjusting for baseline characteristics including baseline AFEQT scores, the underestimated group showed a lower improvement in AFEQT-OS scores at 1 year (underestimated, 2.5 [95% CI, −1.6 to 6.7] vs correctly and overestimated patients, 8.4 [95% CI, 7.0-9.9] points; *P* = .01) (eTable 1 in Supplement 1), and associated with lesser improvements in the AFEQT Symptom domain (−3.3 [95% CI, −8.2 to 1.4] vs 7.7 [95% CI, 6.0-9.4] points; *P* < .001).

**Table 3.** Health Status Outcome and Care Provided at 1 Year

Characteristic	No. (%)			*P* value
	Underestimated^a^	Correctly Estimated	Overestimated^b^	
Patients	42 (12.7)	112 (33.9)	176 (53.3)	NA
Care provided during 1 y after registration				
Treatment escalation	20 (47.6)	70 (63.6)	116 (66.3)	.08
Intensification of antiarrhythmic drugs	11 (26.2)	20 (17.9)	27 (15.3)	.25
Catheter ablation	19 (45.2)	69 (61.6)	113 (64.2)	.08
Electrical/pharmacologic cardioversion	4 (9.5)	6 (5.4)	16 (9.1)	.47
AFEQT questionnaire scores				
Missing at follow-up, No. (%)	34 (11.7)	101 (34.8)	155 (53.4)	NA
At 1 y, mean (SD)				
Overall summary	81.2 (17.8)	90.1 (11.4)	85.8 (14.1)	<.001
Symptoms	80.1 (25.0)	91.3 (11.8)	89.3 (14.3)	<.001
Daily activities	78.1 (22.8)	88.9 (16.6)	83.6 (19.0)	.005
Treatment concern	86.5 (15.6)	90.9 (9.9)	86.3 (15.0)	.04
Change during 1 y (95% CI)^c^				
Overall summary	4.5 (0.1 to 8.8)	10.0 (7.6 to 12.4)	7.0 (5.0 to 8.9)	.047
Symptoms	−2.3 (−7.2 to 2.6)	8.4 (5.6 to 11.1)	7.1 (4.8 to 9.3)	<.001
Daily activities	5.3(−0.2 to 10.8)	8.4 (5.3 to 11.5)	4.7 (2.1 to 7.2)	.17
Treatment concern	9.1 (4.4 to 13.8)	13.2 (10.5 to 15.8)	10.1 (7.9 to 12.3)	.14

Abbreviations: AFEQT, the Atrial Fibrillation Effect on Quality-of-Life; NA, not applicable.

^a^ Denotes physician underestimation of the health status of patients with atrial fibrillation.

^b^ Denotes physician overestimation of the health status of patients with atrial fibrillation.

^c^ The results were adjusted for each baseline AFEQT score.

### Care Provided Within 1 Year

Overall, 206 patients (62.4%) had treatment escalation within 1 year of their initial evaluation. Patients whose health status was underestimated by physicians had a lower likelihood of treatment escalation (47.6%) than those whose health status was correctly estimated (63.6%) or overestimated (66.3%) (Table 3). Rates of treatment escalation were similar among patients whose physicians correctly estimated vs overestimated their health status (63.6% vs 66.3%; *P* = .64). Thus, we combined these 2 groups for further comparison. Patients whose physicians underestimated their health status impairment were less likely to receive treatment escalation compared with those whose health status was correctly estimated or overestimated (47.6% vs 65.2%; *P* = .03). After adjusting for patients’ demographic and clinical characteristics, including baseline AFEQT scores, physician underestimation of the health status of patients with AF was independently associated with less-frequent treatment escalation (adjusted odds ratio, 0.43 [95% CI, 0.20-0.90]; *P* = .02) (eTable 2 in Supplement 1).

## Discussion

Accurately estimating the health status of patients with AF is a foundational requirement for offering treatments to optimize patients’ symptoms, function, and quality of life. In this multicenter, cross-sectional sample of patients with newly recognized AF, we found that physician underestimation or overestimation of the health status of patients with AF was common in routine clinical practice. Physician underestimation was associated with less-frequent treatment escalation for AF and smaller 1-year improvements in patients’ health status. These data highlight an important gap in practice and suggest that the implementation of patient-centered outcome measures into routine clinical care may support more accurate assessments of patients’ health status so that intensification of treatment can be more consistently aligned with the patients’ symptoms of AF. Future studies of the implementation of the AFEQT questionnaire or other health status measures are needed to evaluate whether they can improve care and outcomes.

This cohort study builds on earlier work defining discrepancies between physicians’ and patients’ health status assessments for conditions such as ischemic heart disease and heart failure^12,13,14^ by examining this phenomenon in patients with AF. Our previous study^5^ found that 27% of patients with impaired health status (eg, AFEQT score <80) were noted by physicians as asymptomatic and 45% of patients with relatively unimpaired health status (eg, AFEQT score ≥80) were noted by physicians as having symptomatic AF. While this prior report suggested discordance between physician-estimated and patient-reported assessments of health status for AF, a major limitation was the use of dichotomized physician assessments of symptoms based on medical records, thereby oversimplifying the potential variations in symptom severity and potentially introducing misclassification.^5^ Our present report extends the field by using a standardized rating approach for both patients and physicians at the time of each clinic visit, capturing each of 3 key health status domains and using clinically relevant definitions to comprehensively assess for discordance between patient-reported and physician-estimated health status. We found a lower proportion of patients whose physicians underestimated the their health status (12.7%) than in the prior study (27%). These data are concordant with a previous report from 25 US outpatient practices that found that 12.8% of the patients with chronic coronary artery disease had more frequent angina than recognized by their physician.^13^

Despite the negative impact of lower health status in patients with AF^15^ and the availability of multiple treatment options to improve symptoms and quality of life,^16,17^ we found that the decision to escalate treatment is highly dependent on whether the physician accurately recognized patients’ health status. This is clinically reasonable as physicians are responsible for recommending treatment options, and their interpretation plays a pivotal role in the need for additional treatment. Accordingly, it is not surprising that physicians had similar treatment patterns when accurately assessing or overestimating patients’ health status, but less often intensified treatment in instances in which they failed to recognize (ie, underestimated) the degree to which AF was affecting patients’ health status. The reason that physicians were more likely to overestimate patients’ health status is unknown, but we suspect that it may reflect that the patient was referred specifically for AF management and physicians may have been particularly sensitive to even minor health status impairments when evaluating the patients. We found that, although it did not occur as often as overestimation, physician underestimation of the health status of patients with AF was associated with fewer improvements in their health status over the next year. This highlights the importance of accurately recognizing patients’ health status and supports the use of standardized disease-specific instruments as part of routine clinical care for AF. A recent randomized trial conducted in a tertiary heart failure clinic in the US demonstrated that the routine collection and sharing of the Kansas City Cardiomyopathy Questionnaire with physicians for health status was feasible and enhanced the accuracy of clinicians’ assessments of patients’ health status.^14^ Thus, implementation of disease-specific assessments of patients’ health status in AF might have the potential to enhance the accuracy of health status assessments and treatment appropriateness and ultimately improve patients’ outcomes. Moreover, routine assessment of patients’ health status can provide valuable information not only about their baseline condition, but also their response to initial treatments, enabling further subsequent treatment escalation to optimize symptom management, functional capacity, and quality of life. Further studies are warranted to explore the association between use of these patient-administered tools and both patient-physician communication and clinical outcomes.^18,19^

### Limitations

The findings from this study should be interpreted in the context of several limitations. First, we could not exclude that the use of different instruments in quantifying health status may have accounted for some of the discordance observed, despite rigorous considerations. However, the fact that physicians are not able to estimate well the principal domains of the health status of patients with AF underscores the importance of standardizing assessments of patients’ health status in clinical practice. Second, the nonrandomized observational design introduces potential confounding, although it provides the best way to describe current treatment patterns and outcomes. Unmeasured confounding factors, such as depression, frailty, and economic status, may have influenced physicians’ estimations of patients’ health status and treatment escalation. Third, while we examined patients and physicians in Japan, the generalizability of our results to all Japanese cardiology clinics, noncardiologists, or other health care systems and patient populations deserves further study. Fourth, physicians were aware of the study and that they would be asked to estimate patients’ health status, which may have influenced their assessments. However, this should have biased the findings to the null, as physicians may have been more diligent in assessing patients’ health status as part of this study and the discordance reported in this study may be an underestimate of the true discordance in usual care. Fifth, information regarding physician type and experience that might be associated with discordant recognition, including their subspecialty (eg, electrophysiologist and catheter ablation operator), years of experience, and number of outpatients per day, were not collected. Further research, ideally with a larger sample size, is necessary to thoroughly identify and understand systematic biases associated with discordant perceptions between patients with AF and physicians regarding health status. Sixth, a few participants were already receiving antiarrhythmic medications before enrollment, which may have influenced both patients’ health status and physicians’ estimations. Although we restricted participation to newly diagnosed or referred patients undergoing initial evaluation for AF in cardiology clinics to capture pretreatment data, we were unable to determine the timing of antiarrhythmic medication initiation in this small group of patients.

## Conclusions

In this cohort study, physician underestimation of the symptom burden in patients with AF in outpatient practice was common and was associated with less-frequent treatment escalation and smaller improvements in health status over 1 year. Standardizing assessments of patients’ health status using validated disease-specific questionnaires has the potential to improve the accuracy of assessments of patients’ health status, decisions for treatment escalation, and health status gains over time.

## Notes

Supplement 1. **eFigure 1.** Associations Between Averaged Patients’ Raw Response and Calculated AFEQT-Overall Summary Score **eFigure 2.** Distribution of Averaged Patients’ Raw Responses and Physicians’ Estimations Across Three Health Status Domains **eTable 1.** Adjusted Changes in AFEQT Scores Across Groups **eTable 2.** Factors Associated With Treatment Escalation **eAppendix.** Physician Questionnaire

Supplement 2.
Data Sharing Statement
