# Supplementary material for: Physician Estimates and Patient-Reported Health Status in Atrial Fibrillation
Source: JAMA Netw Open. 2024 Feb 23;7(2):e2356693. doi: 10.1001/jamanetworkopen.2023.56693 (PMC10891467; doi:10.1001/jamanetworkopen.2023.56693)
Supplement: Supplement 1. — eFigure 1. Associations Between Averaged Patients’ Raw Response and Calculated AFEQT-Overall Summary Score eFigure 2. Distribution of Averaged Patients’ Raw Responses and Physicians’ Estimations Across Three Health Status Domains eTable 1. Adjusted Changes in AFEQT Scores Across Groups eTable 2. Factors Associated With Treatment Escalation eAppendix. Physician Questionnaire [file jamanetwopen-e2356693-s001.pdf]

Supplementary Online Content

Ikemura N, Kohsaka S, Kimura T, et al. Physician estimates and patient-reported health status in atrial fibrillation. *JAMA Netw Open*. 2024;7(2):e2356693. doi:10.1001/jamanetworkopen.2023.56693

eFigure 1. Associations Between Averaged Patients' Raw Response and Calculated AFEQT-Overall Summary Score

eFigure 2. Distribution of Averaged Patients' Raw Responses and Physicians' Estimations Across Three Health Status Domains

eTable 1. Adjusted Changes in AFEQT Scores Across Groups

eTable 2. Factors Associated With Treatment Escalation

eAppendix. Physician Questionnaire

This supplementary material has been provided by the authors to give readers additional information about their work.

© 2024 Ikemura N et al. *JAMA Network Open.*

eFigure 1.
Associations between averaged patients' raw response and calculated AFEQT-overall summary score

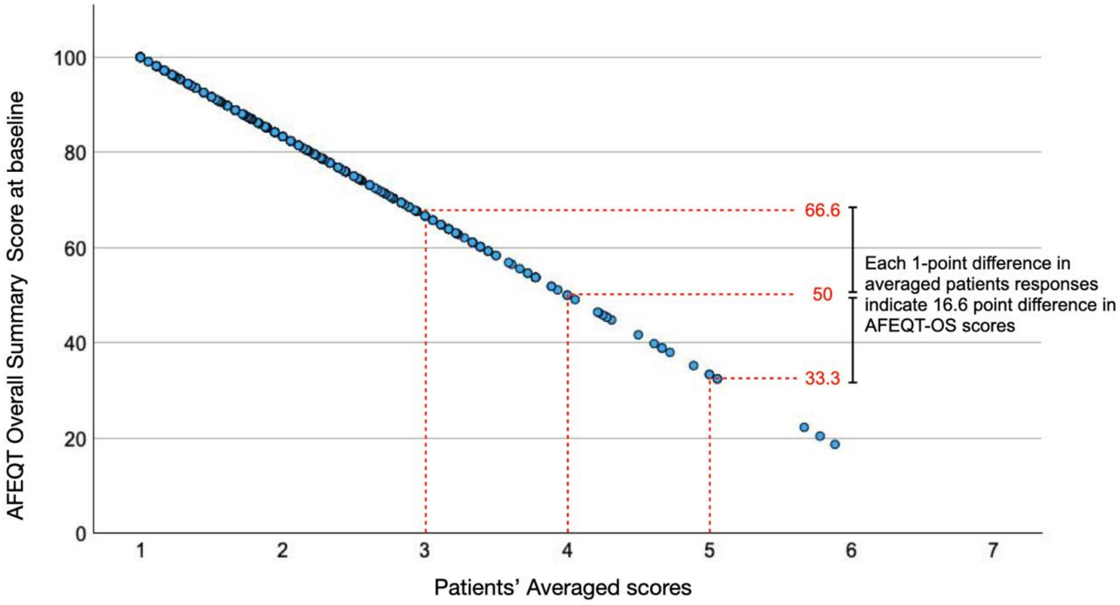

© 2024 Ikemura N et al. *JAMA Network Open.*

eFigure 2.
Distribution of averaged patients' raw responses and physicians' estimations across three health status domains.

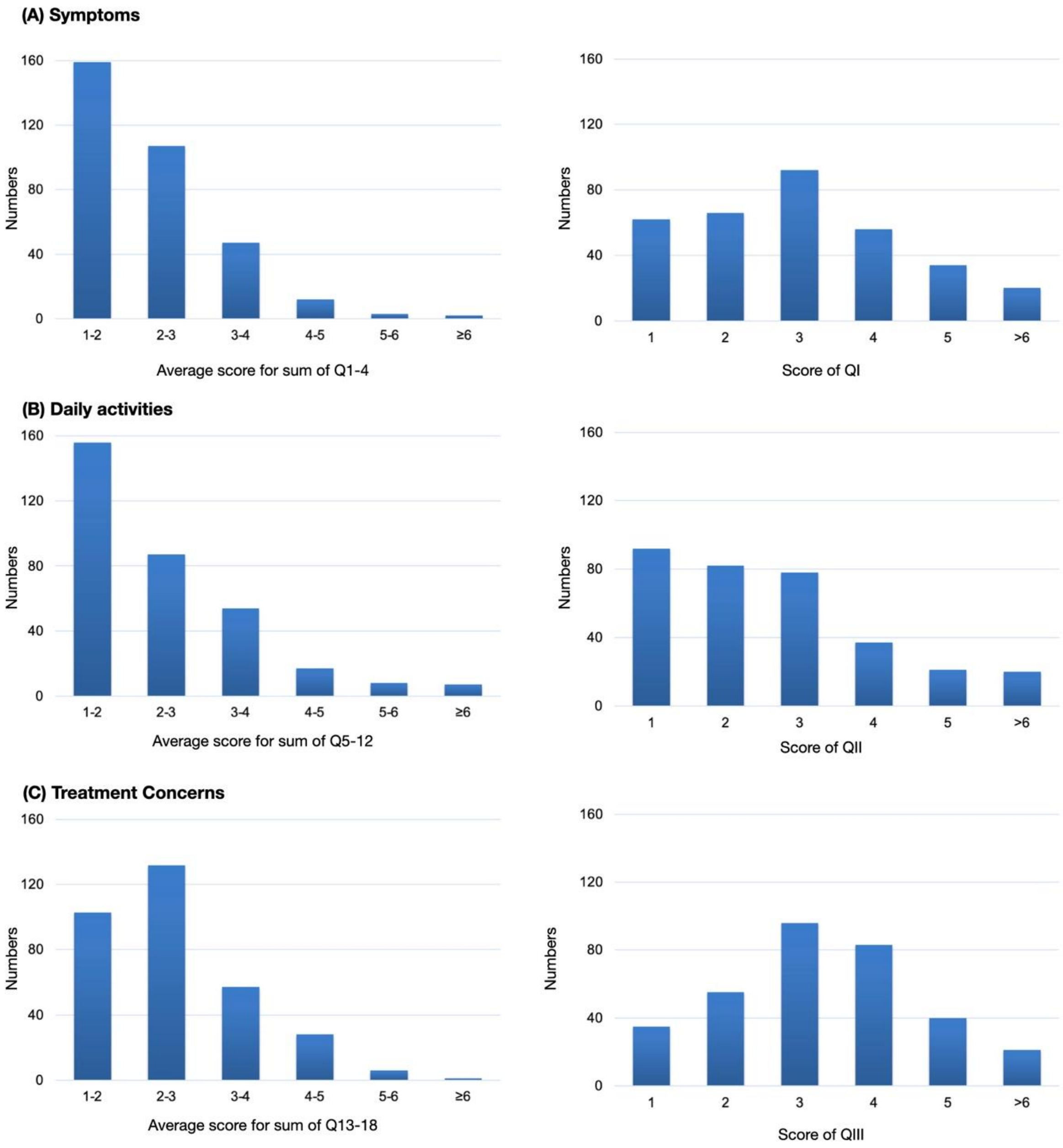

A higher score indicates worse health status.

© 2024 Ikemura N et al. *JAMA Network Open.*

eTable 1. Adjusted changes in AFEQT scores across groups.

AFEQT scores	Estimates	95% confidence interval		P values
		Lower Limit	Upper Limit	
Overall Summary score				
Under estimated group	2.5	-1.6	6.7	.01
Correctly and Over estimated group	8.4	7.0	9.9	
Symptom score				
Under estimated group	-3.3	-8.2	1.4	<0.001
Correctly and Over estimated group	7.7	6.0	9.4	
Daily activities score				
Under estimated group	1.9	-3.4	7.2	.10
Correctly and Over estimated group	6.6	4.8	8.5	
Treatment concern score				
Under estimated group	8.0	3.4	12.5	.15
Correctly and Over estimated group	11.5	9.9	13.1	

The outcome was changes in AFEQT scores, adjusted for site as a random effect to account for within-site correlation and clinically relevant variables as fixed effect, including age (≥75 or not), sex, prior heart failure, type of AF (paroxysmal AF vs. other), use of antiarrhythmic drugs at enrollment, history of catheter ablation for AF, baseline AFEQT-OS score, and CHA_2DS_2-VASc score.

eTable 2. Factors associated with Treatment Escalation.

Variable	Odds Ratio	95% confidence interval		*P* value
		Lower Limit	Upper Limit	
Under-estimation	0.43	0.20	0.90	.02
Age (≥75 or not)	0.80	0.41	1.56	.52
Women	0.93	0.51	1.68	.81
Use of antiarrhythmic drugs at enrollment	0.76	0.39	1.49	.44
Prior catheter ablation for AF	0.36	0.16	0.80	.01
Paroxysmal AF (vs. others)	0.88	0.54	1.43	.60
Clinical history of HF	0.67	0.28	1.59	.36
AFEQT-OS at baseline (per 1-point increase)	0.99	0.97	1.00	.25
CHA_2DS_2-VASc score (per 1-point increase)	0.96	0.77	1.19	.72

Dependent variables: treatment escalation, defined as any of the following: i) intensification of antiarrhythmic drugs (AADs, alternation or the initiation of new medication); ii) ordering electrical or pharmacological cardioversion, or iii) ordering catheter ablation for AF.

© 2024 Ikemura N et al. *JAMA Network Open.*

eAppendix. Physician Questionnaire

Questionnaire for Physicians

Physician Estimation of Patients' Health Status

Patients' Number ________________

Dr's Name ____________________ **Date __________ (YYYY/MM/DD)**

I. How much do you think this patient has been suffering from symptoms of AF for the last four weeks?

Not at all bothered OR he/she did not have symptoms	Hardly bothered	A little bothered	Moderately bothered	Quite a bit bothered	Very bothered	Extremely bothered
1	2	3	4	5	6	7

II. How much do you think this patient's daily activity has been limited by AF for the last four weeks?

Not at all limited	Hardly limited	A little limited	Moderately limited	Quite a bit limited	Very limited	Extremely limited
1	2	3	4	5	6	7

III. How anxious do you think this patient has been about AF and its treatment for the last four weeks?

Not at all bothered	Hardly bothered	A little bothered	Moderately bothered	Quite a bit bothered	Very bothered	Extremely bothered
1	2	3	4	5	6	7

IV. How much do you think symptoms related to atrial fibrillation has affected this patient's daily life for the past four weeks?

1. □ None: Asymptomatic
2. □ Mild: Normal daily activity not affected, symptoms not troublesome to patient
3. □ Moderate: Normal daily activity not affected but patient troubled by symptoms
4. □ Severe: Normal daily activity affected
5. □ Disabling: Normal daily activity discontinued

© 2024 Ikemura N et al. *JAMA Network Open.*
