# Supplementary material for: Physician Estimates and Patient-Reported Health Status in Atrial Fibrillation
Source: JAMA Netw Open. 2024 Feb 23;7(2):e2356693. doi: 10.1001/jamanetworkopen.2023.56693 (PMC10891467; doi:10.1001/jamanetworkopen.2023.56693)
Supplement: Supplement 2. — Data Sharing Statement [file jamanetwopen-e2356693-s002.pdf]

Data Sharing Statement

Ikemura. Physician Estimates and Patient-Reported Health Status in Atrial Fibrillation. *JAMA Netw Open*. Published February 23, 2024. doi:10.1001/jamanetworkopen.2023.56693

Data
Data available: No

Additional Information
Explanation for why data not available: The data and materials used to conduct this research are available to researchers, upon request, for scientific projects aimed at identifying a novel clinical finding that may further improve patient outcomes.
